# Assessment of Meet-URO and CANLPH Prognostic Models in Metastatic RCC: Insights from a Single-Institution Cohort Predominantly Treated with TKIs

**DOI:** 10.3390/diagnostics16030428

**Published:** 2026-02-01

**Authors:** Ömer Faruk Kuzu, Nuri Karadurmuş, Nebi Batuhan Kanat, Dilruba İlayda Özel Bozdağ, Berkan Karadurmuş, Esmanur Kaplan Tüzün, Hüseyin Atacan, Nurlan Mammadzada, Emre Hafızoğlu, Gizem Yıldırım, Musa Barış Aykan, Selahattin Bedir, İsmail Ertürk

**Affiliations:** 1 Cankiri State Hospital, 18100 Çankırı, Turkey; 2 Department of Medical Oncology, Gulhane Research & Training Hospital, 06010 Ankara, Turkey; drnkaradurmus@yahoo.com (N.K.); dilrubaozel@gmail.com (D.İ.Ö.B.); berkankaradurmus@gmail.com (B.K.); esmanurkaplan@hotmail.com (E.K.T.); drhuseyinatacan@gmail.com (H.A.); mammadzadanurlan@gmail.com (N.M.); gizemyildirim_@hotmail.com (G.Y.); musabarisaykan@gmail.com (M.B.A.);; 3 Department of Internal Medicine, Division of Medical Oncology, Gulhane Research & Training Hospital, 06010 Ankara, Turkey; nebibatuhan.kanat@sbu.edu.tr; 4 Department of Medical Oncology, Afyonkarahisar State Hospital, 03030 Afyonkarahisar, Turkey; emrehafizoglu@hotmail.com; 5 Department of Urology, Gulhane Research & Training Hospital, 06010 Ankara, Turkey; selahattin.bedir@sbu.edu.tr

**Keywords:** metastatic renal cell carcinoma, Meet-URO score, CANLPH score, systemic inflammation, prognosis

## Abstract

**Background/Objectives:** Accurate prognostic assessment remains crucial in metastatic renal cell carcinoma (mRCC), especially as treatment options have expanded beyond vascular endothelial growth factor (VEGF)-targeted therapies to include immune checkpoint inhibitors (ICIs) and ICI–TKI combinations. The widely used IMDC classification shows important limitations in the modern therapeutic era, highlighting the need for complementary prognostic tools. In this context, the Meet-URO and CANLPH scores—incorporating clinical, inflammatory, and nutritional markers—have emerged as promising alternatives. To evaluate and compare the prognostic performance of the Meet-URO and CANLPH scoring systems in a real-world mRCC cohort predominantly treated with first-line tyrosine kinase inhibitor (TKI) monotherapy due to limited access to ICI-based combinations. **Methods:** This retrospective single-center study included 112 patients with mRCC. The Meet-URO score was calculated for all patients, while the CANLPH score was assessed in 56 patients with complete laboratory data. CAR, NLR, and PHR were computed using baseline pre-treatment measurements. Overall survival (OS) and progression-free survival (PFS), the latter defined exclusively for first-line therapy, were estimated using the Kaplan–Meier method. Correlations between inflammatory markers and survival outcomes were analyzed using Spearman’s rho. **Results**: Meet-URO demonstrated clear prognostic stratification across all five categories, with the most favorable outcomes in score group 2 and progressively poorer OS and PFS in higher-risk groups. CANLPH also showed meaningful survival discrimination, with the highest inflammatory group (score 3) exhibiting markedly reduced OS and PFS. CAR was the strongest individual predictor of survival, while NLR and PHR showed weaker associations. **Conclusions**: Both Meet-URO and CANLPH provide strong, complementary prognostic information in mRCC, even in a cohort largely treated with TKI monotherapy. Their integration into routine risk assessment may enhance clinical decision-making, particularly in resource-limited settings.

## 1. Introduction

Renal cell carcinoma (RCC) is a clinically heterogeneous and biologically complex malignancy, accounting for 3–5% of all adult cancers and more than 80,000 new cases annually [1]. Approximately one-quarter of patients present with metastatic disease at diagnosis, reflecting its often silent progression and aggressive behavior [2]. Clear-cell RCC is the predominant histopathological subtype, comprising nearly 70% of cases, followed by papillary, chromophobe, and collecting duct variants [3]. While advances in systemic therapy have significantly improved outcomes, survival remains highly variable, highlighting the continued need for accurate and clinically meaningful prognostic tools.

For more than a decade, the International Metastatic Renal Cell Carcinoma Database Consortium (IMDC) classification has served as the most widely implemented prognostic system in metastatic RCC (mRCC), incorporating readily available clinical and laboratory parameters [4,5]. Developed in the era of VEGF-targeted therapies [6], the IMDC model has guided treatment selection and clinical trial stratification with considerable success. However, the rapid evolution of the therapeutic landscape—with the introduction of immune checkpoint inhibitors (ICIs), VEGF–TKI/ICI combinations, and dual immunotherapy—has revealed important limitations of the IMDC score. Notably, favorable-risk patients do not consistently derive an overall survival (OS) benefit from ICI-based combinations [7] and the benefit of first-line nivolumab plus ipilimumab has been confirmed primarily in intermediate- and poor-risk groups [8]. These findings underscore the need for complementary prognostic tools that more accurately reflect contemporary treatment biology.

Systemic inflammation is now recognized as a key determinant of cancer progression and therapeutic resistance across multiple tumor types [9,10]. Simple, inexpensive inflammatory markers derived from peripheral blood—such as neutrophil-to-lymphocyte ratio (NLR), platelet-based indices, and acute-phase reactants—have demonstrated prognostic value across diverse malignancies and therapeutic settings [11,12,13,14,15].

Building upon the IMDC framework and acknowledging the prognostic relevance of systemic inflammation, the Meet-URO score was developed and validated in a cohort of 571 patients receiving second-line nivolumab [16]. By incorporating baseline bone metastases and pretreatment NLR (≥3.2) into the IMDC criteria, the Meet-URO score demonstrated superior prognostic accuracy, both for patients treated with nivolumab and those receiving later-line cabozantinib [16,17].

Traditional molecular biomarkers such as microsatellite instability (MSI) [18] and tumor mutational burden (TMB) [19] have limited relevance in RCC [20], further emphasizing the need for alternative prognostic tools. Recent studies highlight the key role of systemic inflammation—particularly C-reactive protein (CRP)—in shaping outcomes in patients treated with ICIs [21]. In this context, the CANLPH score has emerged as a promising, inflammation-based prognostic model. This composite index incorporates three systemic markers: the CRP-to-albumin ratio (CAR) [22], neutrophil-to-lymphocyte ratio (NLR) and platelet-to-hemoglobin ratio (PHR) [23,24]. Collectively, these parameters reflect systemic inflammation, nutritional status, and hematologic physiology, offering a comprehensive assessment of host–tumor interaction.

Taken collectively, the limitations of traditional prognostic systems, the emergence of novel inflammation-based biomarkers, and the diversity of real-world treatment settings highlight the need for models that maintain prognostic performance across different therapeutic contexts. Notably, the distinct survival gradients observed in our cohort—across both Meet-URO categories and CANLPH inflammatory groups—suggest that integrating clinical and inflammatory markers may offer more robust and clinically actionable prognostic stratification.

Therefore, the present study was designed to comprehensively evaluate and compare the prognostic performance of the Meet-URO and CANLPH scoring systems in patients with metastatic RCC receiving systemic therapy. By assessing these models side-by-side in a real-world cohort, the study aims to determine whether they provide improved and clinically relevant risk discrimination beyond existing frameworks, particularly in settings where access to modern ICI-based combinations remains limited.

## 2. Materials and Methods

This retrospective, single-center observational study included 112 patients diagnosed with renal cell carcinoma (RCC) who received systemic therapy. Clinical, laboratory, and pathological data at the time of diagnosis were extracted from institutional electronic medical records. Among the full cohort, 56 patients had complete biochemical data required for calculation of the CANLPH score, while 112 patients had sufficient information for assessment of the MEET-URO score. Patients lacking one or more laboratory parameters necessary for CANLPH scoring—including C-reactive protein (CRP), albumin, neutrophil count, lymphocyte count, platelet count, and hemoglobin—were excluded from inflammation-based analyses.

All laboratory tests were obtained prior to the initiation of first-line systemic therapy, ensuring that inflammatory markers reflected pretreatment baseline values. Using these measurements, the CAR, NLR, and PHR were calculated for each patient. These indices were used both for constructing the CANLPH score and for additional exploratory analyses. Specifically, Spearman’s rank correlation was applied to evaluate the association of each marker with overall survival (OS) and progression-free survival (PFS) in the available subsets (CAR: *n* = 58 for OS and *n* = 54 for PFS; NLR: *n* = 104 for OS and *n* = 96 for PFS; PHR: *n* = 104 for OS and *n* = 96 for PFS). One-tailed significance testing was employed based on the predefined hypothesis that elevated systemic inflammation would be associated with poorer clinical outcomes. This directional hypothesis was specified a priori based on consistent evidence from prior clinical and biological studies.

Demographic and clinical variables collected included age, sex, histologic subtype, disease stage at diagnosis, and sites of metastasis (bone, liver, lung, central nervous system, lymph nodes, or soft tissue). Treatment-related variables recorded included the number of systemic therapy lines received and the specific first-line regimen administered (sunitinib, pazopanib, cabozantinib, or nivolumab plus cabozantinib).

The CANLPH score was constructed in accordance with the methodology described by Komura et al. [24]. Using cut-off values determined by the Youden Index [25], each of the following was assigned one point: CAR ≥ 1.5, NLR ≥ 2.8, and sex-specific thresholds for PHR (≥2.1 for men and ≥2.3 for women). This yielded a composite inflammation score ranging from 0 to 3 for each patient.

The Meet-URO score incorporates the presence of bone metastases and baseline neutrophil-to-lymphocyte ratio (NLR) ≥3.2 into the IMDC score (a web calculator is available here: https://proviso.shinyapps.io/Meet-URO15_score/, 1 July 2025) [16]. This scoring system stratifies patients from group 1 (most favorable prognosis) to group 5 (poorest prognosis), with lower-numbered groups associated with longer survival. Previous studies have demonstrated that the Meet-URO model outperforms the IMDC model in patients with mRCC treated with second-line nivolumab or cabozantinib, as well as in those receiving first-line nivolumab plus ipilimumab [17,26].

OS and PFS were calculated using standard time-to-event methodology. OS was defined as the interval from diagnosis of metastatic disease to death from any cause or last follow-up. PFS was defined exclusively for first-line systemic therapy as the time from treatment initiation to radiologic or clinical progression or death. Patients without documented progression were censored at their last available disease assessment.

All statistical analyses were performed using the IBM SPSS Statistics 27.0 (IBM Corp., Armonk, NY, USA) software package. Continuous variables were described as medians (interquartile range (IQR)) and categorical variables as percentages. Survival curves and rates were estimated using the Kaplan–Meier method. The log-rank test was used to compare the survival outcomes between the groups. All reported *p*-values were two-sided, and *p*-values < 0.05 were regarded as statistically significant.

## 3. Results

A total of 112 patients were included in the analysis. Baseline demographic, pathological, and clinical characteristics are presented in Table 1. The cohort comprised 37 females (33.0%) and 75 males (67.0%). Clear cell renal cell carcinoma was the predominant histologic subtype (89.3%), followed by papillary (6.3%), chromophobe (1.8%), and collecting duct carcinoma (1.8%). At diagnosis, the majority of patients presented with advanced disease, with 65.2% classified as Stage IV. The most common metastatic sites were the lung (59.8%) and lymph nodes or soft tissue (60.7%), followed by bone (43.8%), liver (22.3%), and the central nervous system (8.9%). Regarding treatment exposure, 62.5% of patients had received three or more lines of systemic therapy. Sunitinib (48.2%) and pazopanib (45.5%) were the most frequently used first-line regimens, whereas cabozantinib (4.5%) and the nivolumab plus cabozantinib combination (1.8%) were less common (Table 1). Given the limited use of immune checkpoint inhibitors (ICIs) in the first-line setting, we also summarized subsequent treatment exposure descriptively: a subset of patients received ICIs in second-line or later lines; however, due to the retrospective design and non-uniform documentation of treatment sequencing, these data were not consistently available for robust risk group-specific stratification.

### 3.1. Survival Outcomes According to the MEET-URO Score

**Figure 1:**
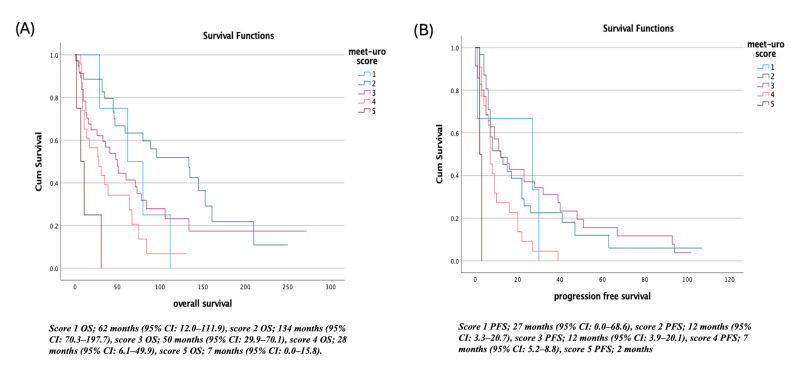
Meet-uroscore (**A**) overall survival, (**B**) progression free survival.

Survival analyses demonstrated a clear separation across MEET-URO risk categories. In the lowest-risk group (score 1), the median OS was 62 months (95% CI: 12.0–111.9), and PFS was 27 months (95% CI: 0.0–68.6). Patients with score 2 showed the most favorable OS at 134 months (95% CI: 70.3–197.7), while PFS was 12 months (95% CI: 3.3–20.7). In the intermediate-risk group (score 3), OS declined to 50 months (95% CI: 29.9–70.1) and PFS to 12 months (95% CI: 3.9–20.1). Higher-risk categories demonstrated progressively poorer outcomes: score 4 was associated with an OS of 28 months (95% CI: 6.1–49.9) and PFS of 7 months (95% CI: 5.2–8.8), while the highest-risk category (score 5) showed the shortest survival, with an OS of 7 months (95% CI: 0.0–15.8) and PFS of 2 months (Figure 1).

### 3.2. Survival Outcomes According to the CANLPH Score

**Figure 2:**
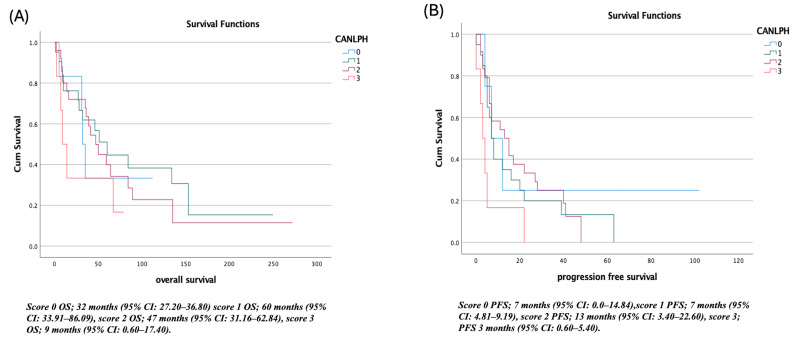
CANLPH score (**A**) overall survival, (**B**) progression free survival.

Similar stratification was observed when patients were categorized according to the CANLPH score. Patients in the lowest-risk group (score 0) had a median OS of 32 months (95% CI: 27.20–36.80) and PFS 7 months (95% CI: 0.0–14.84). The score 1 group demonstrated the longest OS at 60 months (95% CI: 33.91–86.09), while PFS remained 7 months (95% CI: 4.81–9.19). For patients in the score 2 group, OS was 47 months (95% CI: 31.16–62.84) with the longest PFS observed at 13 months (95% CI: 3.40–22.60). The highest-risk group (score 3) exhibited markedly inferior outcomes, with OS falling to 9 (95% CI: 0.60–17.40) months and PFS to 3 months (95% CI: 0.60–5.40) (Figure 2).

### 3.3. Correlation Between Inflammatory Markers and Survival Outcomes

**Figure 3:**
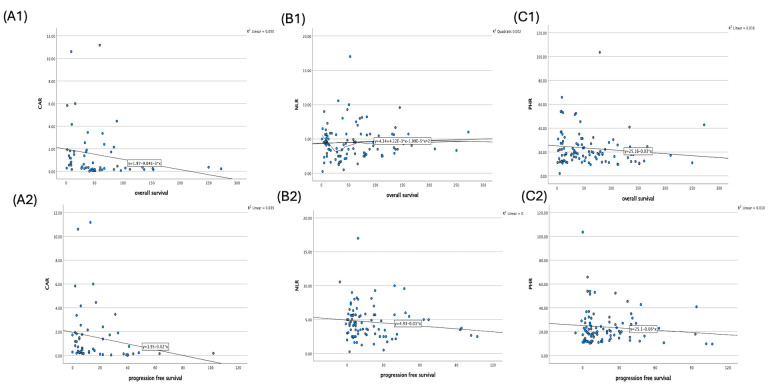
Scatter plots showing the associations between inflammatory markers with survival outcomes: (**A1**) CAR vs. OS, (**A2**) CAR vs. PFS survival; (**B1**) NLR vs. OS, (**B2**) NLR vs. PFS; (**C1**) PHR vs.OS, (**C2**) PHR vs. PFS.

Spearman’s correlation analysis revealed heterogeneous associations between the inflammatory biomarkers and survival endpoints (Table 2). CAR demonstrated significant negative correlations with both OS (ρ = −0.380, *p* = 0.002, *n* = 58) and PFS (ρ = −0.376, *p* = 0.003, *n* = 54), indicating that higher baseline CAR values were associated with shorter survival. In contrast, NLR was not significantly correlated with OS (ρ = 0.041, *p* = 0.341, *n* = 104) or PFS (ρ = −0.149, *p* = 0.074, *n* = 96). PHR demonstrated a weak but statistically significant negative correlation with OS (ρ = −0.179, *p* = 0.035, *n* = 104), while its relationship with PFS did not reach significance (ρ = −0.047, *p* = 0.324, *n* = 96). These findings are illustrated in Figure 3.

## 4. Discussion

The management of mRCC continues to evolve rapidly, driven by advances in systemic therapies and an improved understanding of tumor biology. Prognostic stratification remains essential for optimizing treatment selection, designing clinical trials, and counseling patients about expected outcomes. Historically, the IMDC score has served as the standard prognostic model for patients receiving VEGF-targeted therapies [4,5,6]. However, with the emergence of ICIs, VEGF–TKI/ICI combinations, and dual checkpoint blockade, the limitations of the IMDC system have become increasingly pronounced. Multiple studies have demonstrated that patients classified as favorable-risk by IMDC may not derive a clear OS benefit from ICI-based combinations [7]. Additionally, the confirmed benefit of nivolumab plus ipilimumab in the CheckMate-214 trial was predominantly restricted to intermediate- and poor-risk groups [8], highlighting the need for refined prognostic tools capable of reflecting the biology and treatment response patterns of the modern therapeutic landscape.

The Meet-URO score was developed to address some of these gaps by integrating two additional variables—baseline bone metastases and pretreatment NLR—into the traditional IMDC framework [16]. Both variables have strong biological rationale: bone metastases reflect aggressive disease biology and niche-mediated tumor support, while NLR represents systemic inflammation and dysregulated immunity, both of which are known to influence response to immunotherapy [9,10,11,12,13,14,15]. Validation studies across multiple cohorts have demonstrated that Meet-URO outperforms the IMDC model in predicting OS and PFS in patients treated with second-line nivolumab, cabozantinib, and even first-line nivolumab plus ipilimumab in expanded-access programs [16,17,26]. These results suggest that Meet-URO offers broader prognostic applicability across multiple treatment lines and therapeutic classes.

Our findings strongly align with the existing literature and further validate the prognostic performance of the Meet-URO scoring system. A clear and clinically meaningful separation in survival outcomes was observed across all five Meet-URO categories. Patients in score group 2—representing the most favorable profile within the non-IMDC favorable-risk population—experienced the longest survival, whereas outcomes declined progressively toward the highest-risk group (score 5), which demonstrated markedly inferior OS and PFS. The preservation of this stratification effect in our patient population is particularly noteworthy, given the distinctive treatment patterns in our cohort.

A distinctive aspect of our study is the real-world therapeutic context in which these prognostic systems were evaluated. Unlike high-income countries where ICI–TKI combinations constitute the standard first-line therapy, the vast majority of our patients received single-agent VEGFR-TKIs (mainly sunitinib or pazopanib) in the first-line setting. Limited national access to immunotherapy, financial constraints, and reimbursement restrictions—all common features of developing healthcare systems—loom as major determinants of treatment choice. Despite these constraints, Meet-URO maintained its strong discriminatory performance, reinforcing the robustness and generalizability of this scoring system beyond immunotherapy-rich environments. This finding has meaningful implications for global oncology, as it supports the utility of Meet-URO in diverse socioeconomic and therapeutic settings.

Alongside the Meet-URO score, the CANLPH score also demonstrated significant prognostic relevance in our cohort. Inflammation and nutrition-related biomarkers have increasingly gained attention as prognostic tools in cancer due to their correlation with tumor progression, host immune response, and treatment resistance [21,22,23,24]. The CANLPH model incorporates three readily available laboratory measures—CAR, NLR, and PHR—that together capture systemic inflammation, nutritional status, and hematologic physiology. Consistent with previous findings by Komura et al. [24], we observed that patients in the highest CANLPH risk group (score 3) experienced markedly poorer OS and PFS, whereas survival differences among lower-risk groups (scores 0–2) were less linear, suggesting that CANLPH may be particularly useful for identifying patients with high inflammatory burden rather than for fine prognostic discrimination across all risk strata. CANLPH 0–1 groups exhibited more favorable outcomes, whereas CANLPH 3—the highest inflammatory burden—was associated with a dramatically reduced survival.

Among the individual biomarkers, CAR emerged as the strongest predictor of both OS and PFS. Elevated CAR reflects increased CRP (a marker of cytokine-driven inflammation) combined with reduced albumin (a surrogate for malnutrition and systemic metabolic stress). This dual representation of inflammation and nutritional decline has been shown to predict poor outcomes in numerous malignancies and across treatment modalities [21,22]. Meanwhile, NLR and PHR showed weaker and more heterogeneous associations with survival, which may reflect their sensitivity to transient physiological changes or heterogeneous disease dynamics. Nonetheless, when integrated into the CANLPH model, these markers collectively produced a robust stratification pattern, supporting the clinical value of composite inflammatory scoring systems.

In this study, IMDC served as the reference prognostic model in metastatic renal cell carcinoma. The Meet-URO score, which partially incorporates IMDC variables, showed improved prognostic separation in our cohort, suggesting potential added value from additional clinical features. In contrast, CANLPH reflects a distinct approach based on inflammatory and nutritional status. This difference may indicate a complementary role for CANLPH in patients whose IMDC risk categories do not fully capture systemic conditions. Overall, while IMDC remains a standard benchmark, both models may offer incremental prognostic information within this cohort.

The complementary prognostic performance of Meet-URO and CANLPH in our study highlights the relevance of integrating clinical, metastatic, and inflammatory characteristics into contemporary prognostic assessment. While Meet-URO incorporates tumor burden and immune-inflammatory interactions through bone metastases and NLR, CANLPH focuses more specifically on systemic inflammation and nutritional physiology. The strong prognostic gradients observed with both models underscore the multidimensional nature of mRCC biology and the potential advantage of utilizing more than one scoring system to achieve precise prognostication. Treatment heterogeneity across first-line and subsequent systemic therapies may have influenced the observed survival outcomes within prognostic strata, and these findings should therefore be interpreted in the context of varying treatment exposures. Additionally, the non-linear survival patterns observed in some intermediate prognostic groups should be interpreted with caution, as they may reflect limited sample sizes, treatment heterogeneity, or other unmeasured confounding factors rather than true biological differences. Clinically, Meet-URO and CANLPH may be best viewed as complementary prognostic tools aimed at risk stratification and patient counseling, rather than instruments for routine treatment decision-making.

## 5. Limitations

This study has several limitations that must be considered. First, the retrospective and single-center design increases the potential for selection and information bias. Second, although 112 patients were included overall, only 56 had complete biochemical data required for CANLPH scoring, which may have reduced the statistical power of analyses involving inflammatory biomarkers. This limitation should be explicitly considered when interpreting the comparative prognostic performance and survival gradients observed between CANLPH and Meet-URO, as missing laboratory data may have affected the robustness of these comparisons. Third, treatment heterogeneity—including predominant first-line TKI monotherapy due to restricted access to ICI-based combinations—may affect survival outcomes and limit comparability with international cohorts. Moreover, detailed risk group-specific reporting of second line and later ICI exposure was limited by heterogeneous treatment sequencing and incomplete longitudinal treatment records, precluding reliable stratified analyses without introducing potential selection bias. Finally, incomplete and non-uniform documentation of surgical interventions, including cytoreductive nephrectomy or metastasectomy, limited our ability to evaluate their potential impact on survival outcomes.

The absence of external validation also limits the generalizability of our findings.

## 6. Future Directions

Future research should incorporate multicenter prospective studies involving larger patient populations and including those treated with modern first-line ICI–TKI combinations or dual checkpoint inhibitor regimens. Evaluating dynamic changes in inflammation-related biomarkers during treatment could provide additional prognostic information and support adaptive therapeutic strategies. Integrating established clinical models such as IMDC and Meet-URO with inflammatory or nutritional indices like CANLPH, as well as emerging biomarkers—radiomics, circulating tumor DNA, cytokine signatures, and machine-learning-based risk calculators—may further refine prognostic precision. Given the global disparities in access to immunotherapy, additional real-world studies from low- and middle-income countries are essential to ensure broad applicability and equity in prognostic assessment.

## 7. Conclusions

In conclusion, our findings suggest that both the Meet-URO and CANLPH scoring systems provide clinically relevant and complementary prognostic information in metastatic RCC, even in a real-world population in which most patients received TKI monotherapy rather than modern immunotherapy-based combinations. Meet-URO was able to stratify risk across its categories, while CANLPH identified meaningful differences in survival associated with systemic inflammation and nutritional status. However, given the retrospective design and the absence of external validation, these results should be interpreted with caution. Further prospective, multicenter studies in diverse treatment settings are warranted to confirm and refine the clinical utility of these prognostic models.

## Figures and Tables

**Table 1:** Baseline Clinical Characteristics.

Variable	Category	*n*	%
Sex	Female	37	33.0
	Male	75	67.0
Histology	Clear cell	100	89.3
	Papillary	7	6.3
	Chromophobe	2	1.8
	Collecting duct	2	1.8
Stage at diagnosis	Stage I	8	7.1
	Stage II	24	21.4
	Stage III	7	6.3
	Stage IV	73	65.2
Metastasis	Bone	49	43.8
	Liver	25	22.3
	Lung	67	59.8
	CNS	10	8.9
	LN/Soft Tissue	68	60.7
Systemic therapy lines	≤2	42	37.5
	≥3	70	62.5
First-line treatment	Sunitinib	54	48.2
	Pazopanib	51	45.5
	Cabozantinib	5	4.5
	Nivolumab + Cabozantinib	2	1.8

**Table 2:** Associations of CAR, NLR, and PHR with survival outcomes based on Spearman’s rho coefficients.

Inflammatory Marker	Outcome	Spearman’s Rho	Significance (1-Tailed)	*N*
CAR	OS	−0.380	0.002	58
	PFS	−0.376	0.003	54
NLR	OS	0.041	0.341	104
	PFS	−0.149	0.074	96
PHR	OS	−0.179	0.035	104
	PFS	−0.047	0.324	96

Abbreviations: CAR, C-reactive protein-to-albumin ratio; NLR, neutrophil-to-lymphocyte ratio; PHR, platelet-to-hemoglobin ratio; OS, overall survival; PFS, progression-free survival.

## Data Availability

This manuscript does not report data generation or analysis. Therefore, there are no datasets available for public access.
